# Isoflavones Play a Potential Role in Off-Flavour Scavenging, with a Key Role of IFS2 in Isoflavone Accumulation in Soybean Seeds

**DOI:** 10.17113/ftb.61.04.23.8231

**Published:** 2023-12

**Authors:** Sandeep Kumar, Sagar Banerjee, Amandeep Kaur, Minnu Sasi, Sweta Kumari, Archana Sachdev, Anil Dahuja

**Affiliations:** 1 Division of Biochemistry, ICAR-Indian Agricultural Research Institute, East Patel Nagar, 110012 New Delhi, Delhi, India; 2 Automation and Plant Engineering Division, ICAR-National Institute of Secondary Agriculture, Namkum, 834010 Ranchi, Jharkhand, India; 3 School of Agriculture, Dev Bhoomi Uttarakhand University, 248007 Dehradun, Uttarakhand, India

**Keywords:** isoflavone content, off-flavour formation, lipoxygenase, carbonyl value, thiobarbituric acid value, isoflavone synthase

## Abstract

**Research background:**

Soybean (*Glycine max* (L.) Merr) is a nutrient-rich crop with a high protein content and various bioactive compounds with health-promoting properties. Nevertheless, it is poorly accepted as a food by consumers due to its off-flavour. Due to the ubiquitous presence of isoflavones in soybeans, their inherent antioxidant potential and inhibitory effect on lipoxygenase activity, their sensory properties are currently being considered to mitigate the off-flavour.

**Experimental approach:**

In the present study, the content and composition of isoflavones in 17 soybean cultivars are determined. The correlation between the isoflavone mass fraction and lipid peroxidation was also established, using thiobarbituric acid (TBA) value and carbonyl compound concentration as indices for the development of off-flavour. Cloning, gene expression analysis and *in silico* analysis of isoflavone synthase isoforms (IFS1 and IFS2) were also performed.

**Results and conclusions:**

The total isoflavone mass fraction in soybean genotypes ranged from (153.5±7.2) µg/g for PUSA 40 to (1146±43) µg/g for Bragg. There was a moderately negative correlation between the indices of off-flavour formation and the genistein/daidzein ratio (p<0.1). However, the correlation with total isoflavone mass fraction was found to be insignificant, indicating complex interactions. Higher protein-protein interactions for the predicted structure of IFS2 with other biosynthesis enzymes and its comparatively higher expression in the Bragg than that of IFS1 indicated its more important role in isoflavone synthesis.

**Novelty and scientific contribution:**

The genistein/daidzein mass ratio was found to be an important factor in controlling off-flavour. IFS2 was identified as key to produce soybeans with high isoflavone mass fraction and potentially lower off-flavour formation.

## INTRODUCTION

Soybean is the most widely cultivated legume worldwide. It is primarily used for the extraction of edible oil and as animal feed. The various nutrients in soybean make it an excellent candidate for direct human consumption (*1*). Soy protein is often considered of the highest quality among plant proteins due to its high protein digestibility-corrected amino acid score (PDCAAS), comparable to animal proteins (*2*). Soybean also has a high polyunsaturated fatty acid (PUFAs) content, with a favourable ω-3/ω-6 unsaturated fatty acid ratio. Many other compounds in soybean, like tocopherols (vitamin E), anthocyanins, lecithin and isoflavones, are important for human nutrition (*3*). However, various anti-nutritional factors are also present in the soybean, making it unattractive from a nutritional point of view. It contains a high amount of phytic acid, which binds with divalent metal ions like Fe^2+^ and Zn^2+^, rendering them unavailable for absorption. The development of soybeans with low phytic acid content has been successful in recent studies (*4*). The phytates are essential for phosphate storage and act as feeding deterrents. Therefore, concerns remain regarding their harmful effect on plant survival (*5*).

Beany off-flavour impedes consumer acceptance of soybean and its products (*6*). Lipoxygenase (LOX) (EC 1.13.11.12), an iron-containing dioxygenase, contributes significantly to this astringency. Soybean contains three LOX isoforms with different isoelectric points, optimal pH values and substrate specificity. Of these, LOX-2 is considered responsible for forming off-flavour by catalysing the oxidation of polyunsaturated fatty acids (PUFAs) containing *cis*-1,4-pentadiene structure (*7*, *8*). As soybean has a very high content of PUFAs and LOX, it is highly susceptible to oxidation. LOX generates hydroperoxyl fatty acids by acting with PUFAs. A hydroperoxide lyase (EC 4.2.1.92) converts hydroperoxyl fatty acids into volatile short-chain aldehydes and ketones, producing grassy-beany off-flavour. Inhibition of this off-flavour-producing pathway has been the target of the investigations into reducing off-flavour. An inverse relation between the amount of vitamin E, a potent antioxidant, and thiobarbituric acid (TBA) value, an indicator of the extent of lipid peroxidation, has been observed, suggesting that increased antioxidant content may help improve flavour characteristics (*9*).

Isoflavones are one such class of secondary antioxidant metabolites almost exclusively present in plants belonging to the *Papilionaceae* family, with soybean as the richest source (*10*). Isoflavones impart various health benefits for humans, such as the prevention of osteoporosis and anticancer activity, due to their structural similarity with female estrogens and antioxidant effects (*11*, *12*). Depending on the structure, these properties of different isoflavones vary. Major isoflavones found in soybeans are genistein, daidzein and glycitein. They occur predominantly in the glycosidic forms genistin, daidzin and glycitin, having malonyl and acetyl groups attached to the 6^th^ carbon atom of glucose moiety.

Isoflavone biosynthesis takes place *via* a branch of the phenylpropanoid pathway. Isoflavone synthase (IFS), a cytochrome P450 monooxygenase bound to the endoplasmic reticulum, is a crucial enzyme in this pathway (*13*). This enzyme catalyses hydroxylation coupled with aryl group migration, resulting in shifting of the ring position. This shift in the ring position separates isoflavones from other flavones. In soybean, two isoforms (IFS1 and IFS2) of this enzyme are present (*14*), both of which are suggested to have different functions ranging from roles in stress response to isoflavone biosynthesis in the reserve tissues (*15*).

Reports suggest that isoflavones inhibit the activity of LOX, a principal enzyme involved in the formation of off-flavour. They act by reducing the active state of Fe(III) to the Fe(II) ion and function as antioxidants due to their ability to scavenge free radicals (*8*, *16*). Tewari *et al.* (*17*) reported an increase in the content of isoflavones after treatment with low doses of γ-irradiation. Additionally, they observed a significant decrease in off-flavour indices after γ-irradiation, such as LOX activity, carbonyl concentration and TBA value. The present study focuses on the potential role of isoflavones in preventing off-flavour formation in soybean. The variation in the isoflavone profile of 17 soybean cultivars was measured and their correlation with off-flavour-determining parameters was also calculated. In addition, cloning and gene expression analysis of IFS isoforms in developing soybean seeds of contrasting genotypes was carried out. Moreover, *in silico* analysis was done to determine the differences among IFS isoforms.

## MATERIALS AND METHODS

### Biological material and reagents

Soybean seeds of 17 soybean cultivars were generously provided by Dr SK Lal, Division of Genetics, ICAR-Indian Agricultural Research Institute, New Delhi, India. The genistein and daidzein standards were procured from Sigma-Aldrich, Merck (Burlington, MA, USA). TRIzol and PrimeScript^TM^ 1^st^ strand cDNA synthesis kits were purchased from TaKaRa Bio Inc. (Shiga, Japan). The pENTR^TM^/D-TOPO^TM^ vector was purchased from Thermo Fisher Scientific (Waltham, MA, USA) and the SYBR^R^ Green JumpStart^TM^ Taq ReadyMix^TM^ was bought from Sigma-Aldrich, Merck. Thiobarbituric acid (TBA), 2,4-dinitrophenylhydrazine and other reagents were purchased from Sisco Research Laboratories Pvt. Ltd. (SRL) (Mumbai, India).

### HPLC analysis for determining isoflavone content and composition

Isoflavone mass fraction and composition were determined using HPLC. The sample extract, standard curve and HPLC conditions were prepared as per Kumar *et al.* (*18*) with slight modifications. A mass of 125 mg of ground soybean seeds was weighed and extracted using 5 mL ethanol and 1 mL concentrated H_2_SO_4_ for 2 h in the boiling water bath. The extracts were centrifuged (Heraeus Multifuge X3R; Thermo Fisher Scientific) at 10 000×*g* for 10 min, and the supernatant was neutralised using 10 M NaOH. After syringe filtration (0.45 µm), the samples were injected into the HPLC. The chromatography was performed in a Waters 2695 chromatograph (Waters Corporation, Milford, MA, USA) with a 2998 photodiode array detector and a Phenomenex Luna C_18_ (250 mm×4.6 mm, 5 µm; Torrance, CA, USA) analytical column as the stationary phase. A binary gradient of acetonitrile (ACN) and water was used as the mobile phase with a flow rate of 1.5 mL/min. For the first 1 min of HPLC separation, 13 % ACN was used, with the ACN content reaching 30 % at 20 min and again reducing to 13 % at the end of the run, *i.e.* 25 min. The absorbance was measured at 254 nm using spectrophotometer (EVOLUTION One Plus; Thermo Fisher Scientific) and the relative mass fraction of isoflavones was determined by CSW17 software (Data Apex®, Prague, Czech Republic) and expressed as µg/g of seed dry mass.

### Estimation of TBA value

Malondialdehyde (MDA), a degradation product of lipid hydroperoxides, is produced by lipid peroxidation. The MDA formed in soybean samples was measured as TBA value based on the adduct development after the reaction of MDA with TBA. The adduct gives a pink colour with the absorbance measured at 532 nm (*19*). Approximately 400 mg of soybean seeds soaked overnight were homogenised with 4.0 mL of distilled water, followed by centrifugation at 13 000×*g* for 35 min to obtain the clear extract. Then, 0.2 mL of this extract was mixed with 0.2 mL of 8.1 % sodium dodecyl sulphate, 1.5 mL of 20 % acetic acid (pH=3.5) and 1.5 mL of 0.8 % TBA and kept in a boiling water bath for 60 min. After cooling, 1.0 mL of distilled water and 5.0 mL of *n*-butanol pyridine (15:1) were added, and the tubes were shaken vigorously. The mixture was then centrifuged at 4000×*g* for 10 min. The pink-coloured organic layer formed at the top was separated from the aqueous phase and absorbance was measured at 532 nm. The standard curve was prepared using 1,1,3,3-tetraethoxypropane ranging from 10 to 70 nmol and the concentration of lipid peroxides was expressed in nmol of MDA per g fresh mass.

### Determination of the concentration of carbonyl compounds

The concentration of carbonyl compounds was determined using the method described by Henick *et al.* (*20*). The 2,4-dinitrophenyl hydrazine reacts with the aldehydes and ketones to form their 2,4-dinitrophenylhydrazone derivatives, which are converted to wine red-coloured complexes by KOH under alkaline conditions. The samples were extracted (0.5 mL) in the same manner as for the TBA value, then mixed with 0.5 mL of 2,4-dinitrophenylhydrazine (0.05 %) and heated in a water bath at 60 °C for 30 min. After cooling, 1.0 mL of KOH (4 %) was added, and a characteristic wine-red colour appeared. Absorbance was measured at 480 nm 10 min after the appearance of the wine-red colour and the concentration was determined with the following formula:


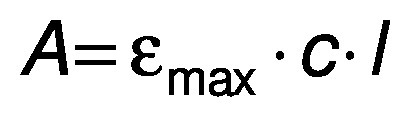
 /1/

where *A* is absorbance, *ε*_max_=2.72·10^4^ L/(mol·cm), *c* is concentration (mol/L) and *l* is path length (cm).

### Quantitative expression analysis of the isoflavone synthase gene

The quantitative real-time gene expression analysis of isomers IFS1 and IFS2 was performed on days 35, 45 and 55 after flowering (DAF) in contrasting soybean genotypes grown in the open field at ICAR-IARI, New Delhi, India. Total RNA was isolated using the TRIzol^®^ method (*21*) and the first strand of cDNA was synthesised from it. All the primers for quantitative polymerase chain reaction (qPCR) (~150 bp) were designed using PrimerBlast (*22*) available at NCBI (http://www.ncbi.nlm.nih.gov/tools/primer-blast/). The housekeeping gene actin 2/7 was used as an internal control for data normalisation (Table S1). The qPCR was carried out using a Bio-Rad CFX96 machine (Bio-Rad, New Delhi, India) in a reaction volume of 20 mL containing SYBR^®^ Green JumpStart^TM^ Taq ReadyMix^TM^ (Sigma-Aldrich, Merck), forward primer (10 mM), reverse primer (10 mM) and 10 ng/mL of cDNA using conditions of 94 °C for 5 min, then 40 cycles at 94 °C for 15 s, 55 °C for 30 s and 72 °C for 45 s. The relative expression level was calculated following the 2^-ΔΔCt^ method (*23*). Standard errors and standard deviations were calculated from three technical and biological replicates. Seed samples 35 DAF were considered the calibrator. The resulting PCR products were also analysed by agarose gel electrophoresis to determine the specificity.

### Cloning of IFS1 and IFS2 cDNA

The total RNA was isolated from soybean seeds using RNAiso plus (TaKaRa Bio Inc.) following the manufacturer's protocol. The full-length IFS1 and IFS2 coding sequences were amplified from the soybean cultivar DS2706. Both forward and reverse primers were designed using PrimerBlast (*22*). The amplified PCR products were eluted from the gel using a Quick Guide PCR purification kit (SolGent, Daejeon, Republic of Korea) and kept for ligation in a pENTR/D-TOPO vector, an entry vector for the Gateway® System, for 30 min at 23 °C in a molar ratio *r*_insert,vector_=2:1. A volume of 3 µL of ligation mix was transformed into TOP10 chemically competent *E. coli* cells, and 6 colonies from each clone were inoculated for plasmid isolation. PvuII restriction digestion and PCR amplification confirmed the cloning of the cDNA sequence. The confirmed clones were subjected to commercial Sanger sequencing (Chromus Pvt. Ltd., Bengaluru, India) to discern their nucleotide sequence.

### In silico analysis

The nucleotide and translated amino acid sequences of isomers IFS1 and IFS2 were compared using NCBI BLAST (https://blast.ncbi.nlm.nih.gov/Blast.cgi accessed on 9 June 2022) set to default parameters (*24*). The three-dimensional structures of the IFS1 and IFS2 proteins from their amino acid sequences were generated using RoseTTAFold (*25*), a deep learning-based structure prediction tool (https://robetta.bakerlab.orgaccessedon4July2022). This nonhomology-based algorithm works by detecting patterns in protein sequences, interactions among the amino acid residues and probable protein tertiary structures, simultaneously determining everything. The Ramachandran plots of predicted models were generated on the RoseTTAFold server, where the favoured rotamers and Ramachandran plot distribution with *φ* and *ψ* angles were analysed (*26*). The interactions of IFS1 and IFS2 with other proteins were studied using STRING (https://string-db.org/ accessed on 11 January 2023) (*27*), a database containing known and predicted protein-protein interactions.

## RESULTS AND DISCUSSION

### Mass fraction and composition analysis of isoflavones in soybean genotypes

The mass fraction and composition of isoflavones in soybean genotypes vary widely (*28*), with malonylated derivatives of isoflavone glucosides, *i.e.* genistin and daidzin being the most abundant (*29*). The health benefits, bioavailability and LOX inhibition potential of various isoflavones could also vary with structural characteristics. Due to the prevalence of these substituted derivatives, these isoflavone glucosides were converted into their corresponding isoflavone aglycones by acid hydrolysis.

**Fig. S1:**
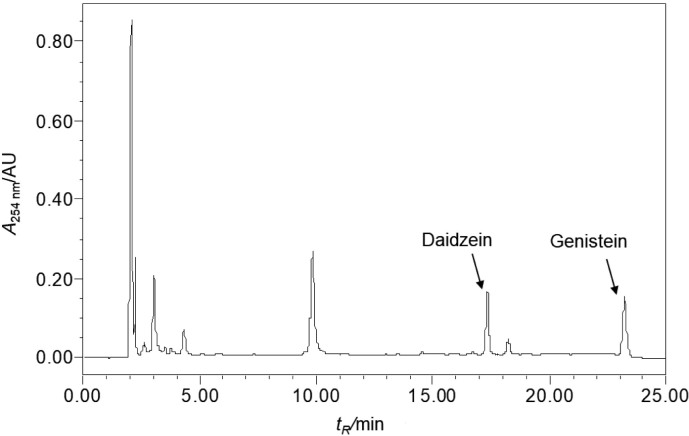
A representative chromatogram of soybean cultivar (PK1042) for isoflavone estimation. Peaks corresponding to major isoflavones, *i.e.* daidzein and genistein, are labelled

Seventeen different soybean genotypes were analysed for total isoflavone content and composition to investigate their relationship with indices of off-flavour development in soybeans. Enormous differences in the total isoflavone content and the mass fraction of individual forms of these isoflavones were found among cultivars (Table 1 and Fig. S1). The total isoflavone mass fraction was expressed in μg/g of soy flour, ranging from (153.5±7.2) µg/g for PUSA 40 to (1146±44) µg/g for Bragg. Bragg and PUSA 40 were identified as the soybean genotypes with high and low isoflavone mass fraction, respectively, and were selected for gene expression analysis of isoflavone synthase (IFS). Similar studies elsewhere have also found enormous differences in isoflavone content and composition among soybean genotypes grown in different ecoregions (*28*). PK 1042 and PK 416 were identified as the soybean genotypes with a maximum (1.64) and minimum (0.33) genistein/daidzein mass ratio, respectively. This is significant as genistein is the isoflavone with higher antioxidant activity than daidzein (*16*, *30*). The analysed genotypes showed enormous variation in both isoflavone content and composition, which can prove useful for developing isoflavone-rich soybean varieties through breeding programmes.

**Table 1:** Isoflavone mass fraction and composition of 17 soybean genotypes

**Cultivar**	***w*/(µg/g)**			***m*(genistein/*m*(daidzein)**
Genistein	Daidzein	Total Isoflavones
**PK 1042**	(411±43)^c^	(662±20)^a^	(1072±60)^b^	1.61
**BRAGG**	(600±33)^b^	(546±12)^b^	(1146±44)^a^	0.91
**PUSA 16**	(159.3±8.6)^i^	(79.0±3.8)^k^	(238±12)^l^	0.5
**PUSA 22**	(212.8±3.9)^g^	(118.9±5.3)^j^	(331.7±9.2)^jk^	0.56
**PUSA 24**	(333±28)^de^	(244.7±2.3)^e^	(578±27)^e^	0.73
**PUSA 37**	(185.19±9.69)^ghi^	(150.88±2.3)^hi^	(336±11)^ij^	0.81
**PUSA 40**	(108.4±3.2)^j^	(45.1±4.2)^l^	(153.5±7.2)^m^	0.42
**PUSA 9814**	(246.8±3.9)^f^	(156.8±2.9)^h^	(403.4±5.9)^h^	0.64
**PK 416**	(302±30)^e^	(91.3±8.2)^k^	(393±22)^h^	0.3
**SL 525**	(625±59)^a^	(378±15)^c^	(1003±68)^c^	0.6
**BS 1**	(276.9±2.8)^f^	(371±32)^c^	(648±34)^d^	1.34
**AMSS 34**	(248.2±2.7)^f^	(118.0±10.4)^j^	(366±12)^i^	0.48
**EC 109514**	(263±14)^f^	(318.0±4.0)^d^	(581±18)^ef^	1.21
**EC 114526**	(329±40)^d^	(203.7±3.7)^f^	(533±43)^f^	0.62
**UPSL 340**	(176.5±6.8)^hi^	(124.8±4.4)^j^	(301±11)^k^	0.71
**UPSL 785**	(272.0±6.3)^f^	(179.2±5.7)^g^	(451.21±1.7)^g^	0.66
**UPSL 19**	(201.1±6.1)^gh^	(150.6±6.1)^i^	(351.8±4.0)^ij^	0.75

The results are presented as mean value±standard deviation (*N*=3). Duncan's multiple range tests (DMRT) were performed to check the significant difference for the isoflavone mass fraction at the 5 % level of significance (p<0.05), where the values denoted by the same letter in superscript belong to the same group and thus the difference among them is statistically insignificant

### Assessment of lipid peroxidation in different soybean genotypes

**Fig. 1:**
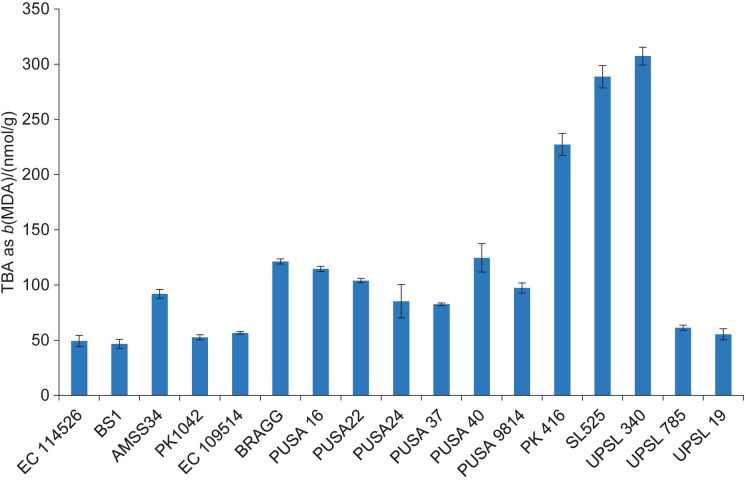
Determination of thiobarbituric acid (TBA) value (index of lipid peroxidation) in 17 soybean genotypes. The results are presented as mean value±standard deviation (*N*=3)

**Fig. 2:**
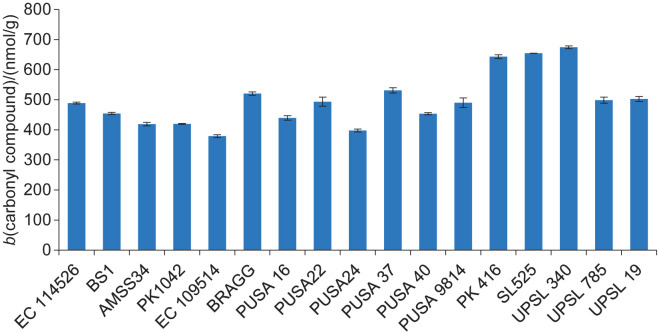
Concentration of carbonyl compounds determined in 17 soybean genotypes. The results are presented as mean value±standard deviation (*N*=3)

The TBA value is a well-established measure of lipid peroxidation (*31*). It expresses the lipid hydroperoxides formed during oxidation in nmol per g of seed. The TBA value in soybean genotypes ranged from (46.7±4.2) nmol/g for BS1 to (307.3±8.1) nmol/g for UPSL 340 (Fig. 1). Similarly, the carbonyl value measures the amount of aldehydes and ketones associated with the formation of off-flavours. Its values were expressed in nmol per g of seed and ranged from (379±5) nmol/g for EC 109514 to (674±5) nmol/g for UPSL 340 (Fig. 2). The extent of correlation between the isoflavone mass fraction and the parameters used as indices of off-flavour was measured using Pearson's correlation coefficients. The observed value for the correlation coefficient between the total isoflavone content and the carbonyl value was 0.089, and that of the TBA value and total isoflavone content was 0.016 (all statistically insignificant at p<0.1) (Table 2). As mentioned above, genistein has been reported to have the highest antioxidant activity among the various soy isoflavones. Therefore, the correlation of particular forms of isoflavones with these parameters is expected to be different. Hence, the correlation coefficients of the parameters determining off-flavour were also calculated for the genistein and genistein/daidzein mass ratio. The correlation between the genistein content and the carbonyl value was -0.12, and the correlation between the TBA value and the genistein content was -0.15 (not statistically significant at p<0.1). In contrast, the correlation coefficients for the genistein/daidzein mass ratio for the TBA and carbonyl values were -0.41 and -0.42, respectively, indicating a weak but statistically significant correlation (as both values are statistically significant at p<0.1 but not significant at p<0.05). The higher antioxidant potential of genistein than daidzein can be the reason for this observation (*32*). Genistein is also a more potent inhibitor of soybean LOX, with a half inhibitory concentration (IC_50_) value of 107 µM compared to 140 µM for daidzein (*16*, *33*). Previously, Dahuja and Madaan (*9*) had also observed an inverse relationship between the contents of antioxidant enzymes and parameters determining off-flavour such as TBA and carbonyl values. Therefore, the ability of genistein to inhibit LOX and to be a general antioxidant seems to be responsible for a reduction in the indices of off-flavour formation. However, isoflavones themselves can contribute to bitterness by binding to taste receptors (*34*–*36*). Since the beany off-flavour is mainly caused by lipid peroxidation, a delicate balance must be achieved in the quantity and type of antioxidants.

**Table 2:** Correlation coefficients between different variables, namely isoflavones, genistein, genistein/daidzein (G/D) mass ratio, carbonyl value and thiobarbituric acid (TBA) value in soybean genotypes

**Parameter**	Isoflavone	Genistein	G/D	Carbonyl value
**Genistein**	0.948 (0.000)*			
**G/D**	0.593 (0.012)*	0.80 (0.733)		
**Carbonyl value**	0.089 (0.733)	-0.120 (0.646)	-0.415* (0.097)	
**TBA value**	0.02 (0.951)	-0.158 (0.545)	-0.414* (0.099)	0.764* (0.000)

The values in parentheses represent the respective p-values of correlation coefficients. *Statistically significant at p<0.1

### Quantitative gene expression analysis of isoflavone synthase and isoflavone content in three stages of seed development

**Fig. 3:**
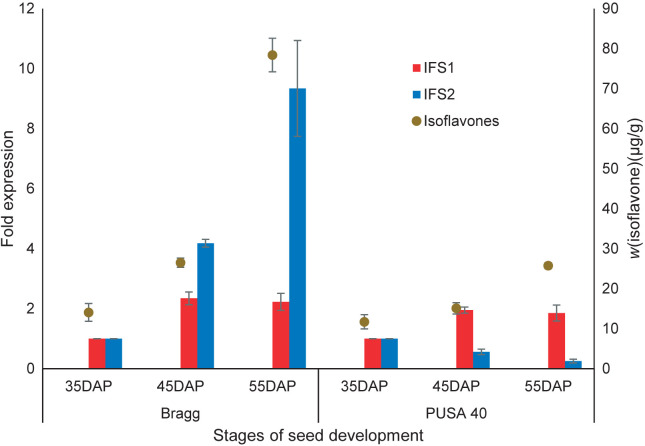
Variation in isoflavone synthase (isomers IFS1 and IFS2) gene expression and corresponding isoflavone accumulation in contrasting (Bragg=high isoflavone and PUSA 40=low isoflavone mass fraction) soybean genotypes in three stages of seed development. The results are presented as mean value±standard deviation (*N*=3)

Isoflavone synthase (EC 1.14.14.87) is the key enzyme of isoflavone biosynthesis responsible for the synthesis of isoflavone aglycone backbones. There are two different isoforms of IFS in soybean. To determine the relative contribution of these isoforms to isoflavone accumulation in soybean seeds, we carried out gene expression analysis of IFS1 and IFS2 in two contrasting soybean genotypes, Bragg (high isoflavone content) and PUSA 40 (low isoflavone content) at three stages of seed development. Expression profiling suggests that the expression level of IFS1 increased with the progress of the developmental stage in PUSA 40 (Fig. 3). However, a nonlinear and irregular trend in the expression of IFS1 was observed in Bragg; it first increased 2.34-fold from 35 to 45 days after flowering (DAF) and then decreased 2.22-fold until 55 DAF. An increasing trend of IFS2 expression was observed in Bragg, but the expression level decreased with each developmental stage in PUSA 40. In quantitative terms, an overall 9.34-fold increase in IFS2 expression was observed in Bragg on 55 DAF compared to its expression on 35 DAF. In comparison, the gene expression in PUSA 40 was reduced to approx. 6 % of the value on 35 DAF.

The corresponding mass fractions of the total isoflavone in each stage were also determined using HPLC. The total isoflavone mass fraction in Bragg remained higher than that in PUSA 40 in all three stages of seed development. In addition, the total isoflavone mass fraction in Bragg increased from 14.07 to 78.4 µg/g on 55 DAF, but in PUSA 40, this increase was only approx. 2.19-fold from 35 DAF to 55 DAF.

Dhaubhadel *et al.* (*37*) observed the accumulation of IFS2 transcripts in the later stages of seed development, but IFS1 expression remained constant throughout. Gutierrez-Gonzalez *et al*. (*15*) also observed a more significant increase in IFS2 expression of up to 20-fold 70 days after pollination compared to 30 days after pollination during seed development. IFS1, in contrast, increased only 4-fold in the same period.

The expression of IFS2 matched the pattern of isoflavone accumulation in developing soybean seeds. However, no such relationship could be observed with the expression levels of IFS1, as the two contrasting cultivars, despite having similar expression levels of IFS1, differed significantly in the isoflavone mass fractions in all the stages of seed development, with the difference in the isoflavone being more pronounced on 55 DAF. Thus, IFS2 may directly affect isoflavone accumulation during the grain-filling stage.

### Cloning and in silico analysis of IFS1 and IFS2

**Fig. 4:**
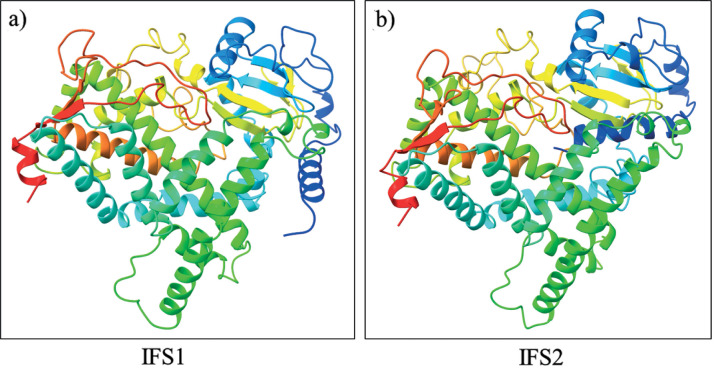
3D structures of isoflavone synthase isomers: a) IFS1 and b) IFS2 generated using RoseTTAFold (*25*). The naringenin (a substrate for genistein formation) is also shown at the binding site of IFS2

**Fig. 5:**
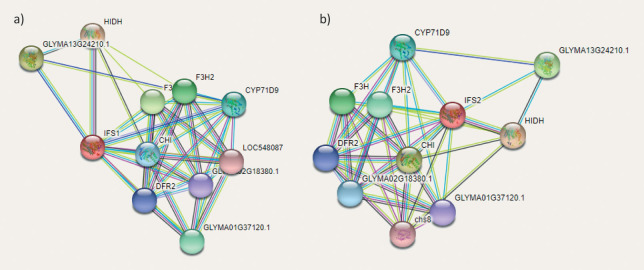
Protein-protein interactions for: a) IFS1, b) IFS2 prepared using STRING (*27*). IFS1=isoflavone synthase 1, IFS2=isoflavone synthase 2, HIDH=2-hydroxyisoflavone dehydratase, GLYMA13G24210.1=uncharacterised (SAM-binding methyltransferase superfamily), F3H=flavanone 3-hydroxylase, F3H2=uncharacterised (flavanone 3-hydroxylase), CHI=chalcone-flavanone isomerase, DFR2=dihydroflavonol-4-reductase, details in Fig. S6

**Fig. 6:**
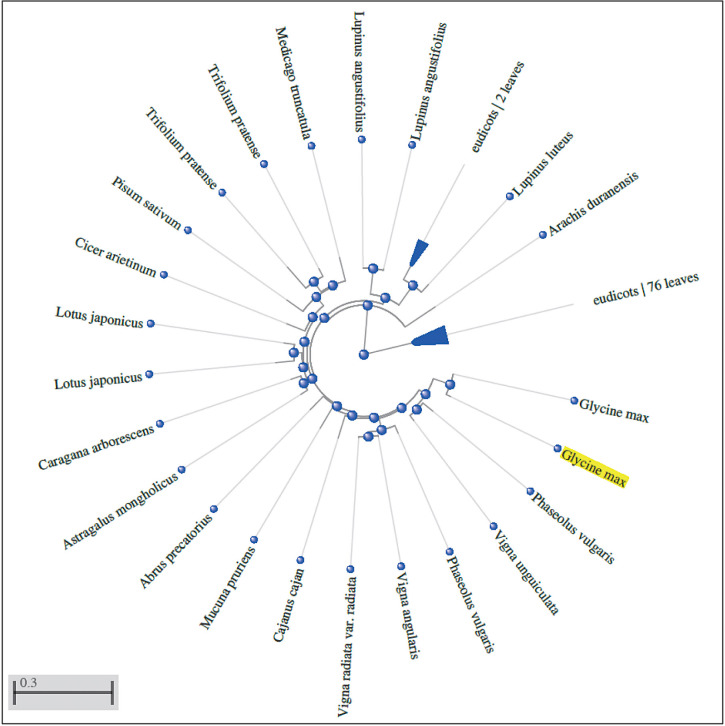
The phylogenetic tree of isoflavone synthase prepared using isoflavone synthase isomer IFS2 as a query. Pairwise BLAST (*24*) and fast minimum evolution method were used to prepare the tree from NCBI's non-redundant (nr) database. Taxonomic names are used as labels

**Fig. S2:**
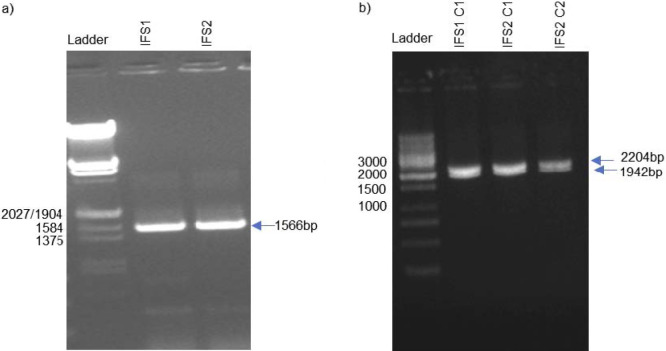
Polymerase chain reaction (PCR) amplification of isoflavone synthase isomers: a) IFS1 and IFS2 transcripts, b) PvuII restriction products (2204 and 1942 bp) of IFS1 and IFS2 cloned into the pENTR-D-TOPO vector

**Fig. S3:**
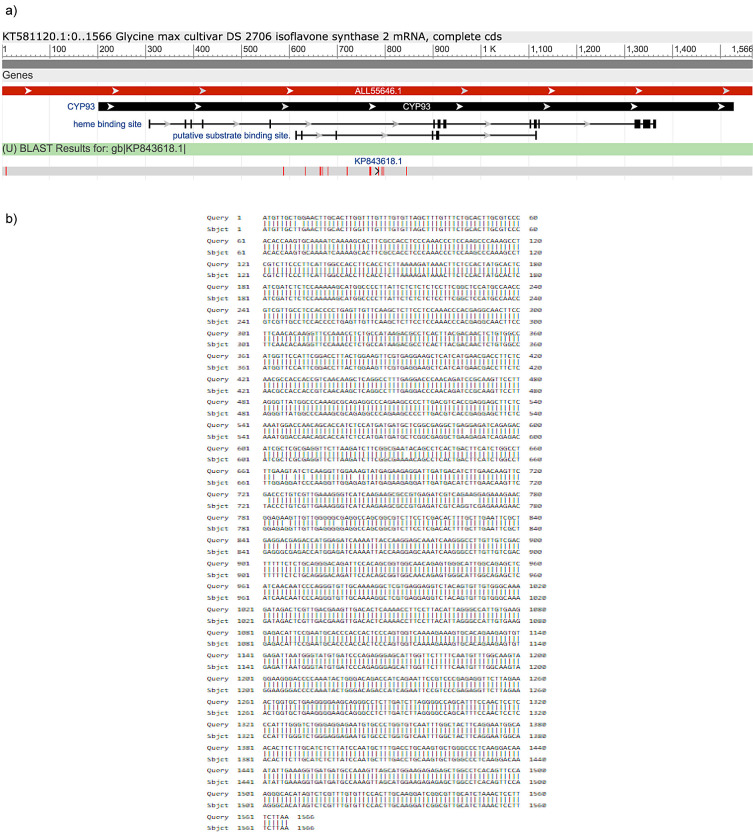
Nucleotide sequence alignment of IFS1 and IFS2 coding sequences showing 99.04 % identity: a) graphical summary of the alignment, and b) sequence alignment with IFS1 as query sequence and IFS2 as subject sequence

**Fig. S4:**
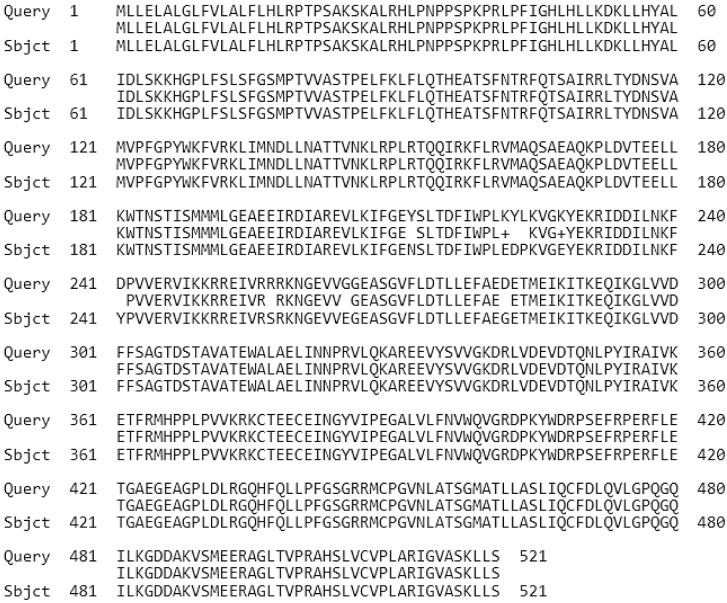
Sequence alignment of isoflavone synthase IFS1 and IFS2 proteins showing 98.27 % identity

**Fig. S5:**
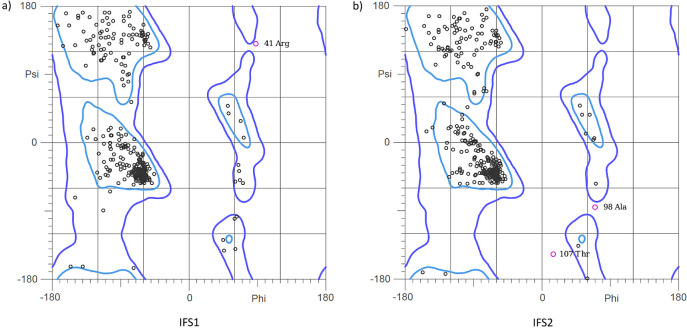
Ramachandran plot generated on the RoseTTAFold (*25*) for isoflavone synthase: a) IFS1 and b) IFS2 3D models

**Fig. S6:**
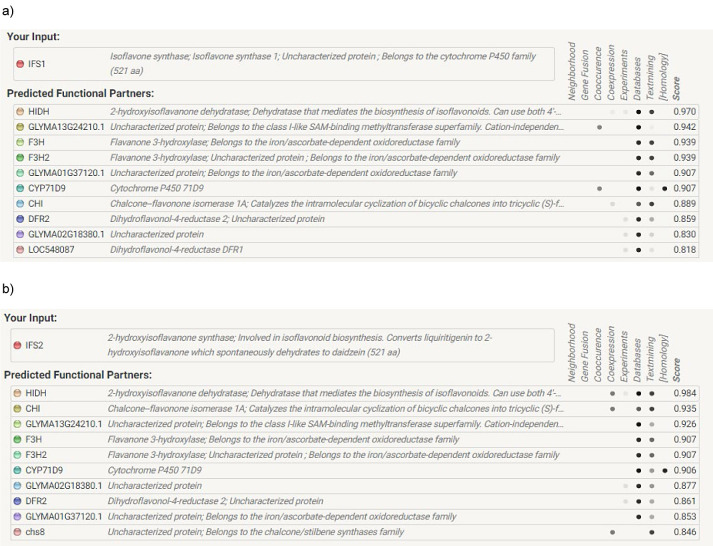
STRING scores for isoflavone synthase isomers: a) IFS1 and b) IFS2 with different proteins

The qRT-PCR and the analysis of isoflavone content indicated a putative role of isoflavone synthase in the accumulation of isoflavones in soybean seeds. Considering this, these cloning and *in silico* analyses of isoflavone synthase isoforms were performed. Specific primers for polymerase chain reaction (PCR) amplification of IFS cDNA genes were designed based on the sequence information available at NCBI (https://blast.ncbi.nlm.nih.gov/Blast.cgi accessed on 9 June 2022). The PCR products were run on an agarose gel, and the approximate size of the amplicon was 1566 bp (Fig. S2) for both IFS1 and IFS2. The obtained PCR products were then eluted from the gel and cloned into the pENTR/D-TOPO vector. The cloning of IFS1 and IFS2 was confirmed by plasmid PCR and restriction digestion (*Pvu*II). The PvuII restriction enzyme has two cut positions (174 and 812) in the TOPO entry vector. We found linear bands of 2204 bp (1566 bp insert+638 bp vector) and a 1942 bp vector backbone in both IFS1 and IFS2. The gene sequences of the cloned products were submitted to the National Center for Biotechnology Information (NCBI) GenBank (Bethesda, MD, USA) under accession numbers KP843618 and KT581120). The 1566 bp long IFS1 and IFS2 coding sequences showed an identity of 99.04 % with difference in 15 nucleotides (Fig. S3). The amino acid sequences of IFS1 and IFS2 were also aligned and showed an identity of 98.27 %, with a difference of 9 out of 521 amino acid residues (Fig. S4). The 3D models of both IFS1 and IFS2 were generated using RoseTTAFold (Fig. 4). The Ramachandran plot for IFS1 and IFS2 showed over 94.8 and 96.5 % of the residues in the most favoured region, respectively (Fig. S5). The IFS isoforms showed minor differences in their tertiary structures. Nevertheless, their predicted interactions with other proteins differed significantly. Interestingly, in the interaction study performed using STRING, IFS2 showed a maximum interaction with chalcone isomerase (CHI) with a score of 0.933 (Fig. 5 and Fig. S6), which was not the case for IFS1. In the phylogenetic tree prepared using the translated IFS2 protein sequence (Fig. 6, highlighted in yellow), the IFS1 sequences were grouped separately, and more than one copy of IFS was observed in many other legume species.

Dastmalchi *et al.* (*38*) studied the interactions and subcellular localisation of the most important enzymes in isoflavone biosynthesis. They found the interaction of chalcone reductase (*Gm*CHR14) with *Gm*IFS2 but not with *Gm*IFS1, which is consistent with the results obtained in this study. The *Gm*CHR14 is upstream of isoflavone synthase in the phenylpropanoid pathway and is responsible for isoflavone biosynthesis. In a recent study, a CRISPR-mediated knockdown of IFS1 was performed and nearly all the physiological processes in the mutants were intact, indicating a role of IFS2 (*39*). However, a curious finding of this study was the greater effect of IFS1 knockdown on genistein rather than daidzein accumulation, which could explain the higher amount of daidzein in Bragg than genistein. In view of this, IFS2 appears to be better suited for the formation of a metabolon (a multienzyme complex), which may lead to efficient and effective substrate channelling and thus to a higher accumulation of isoflavones through this pathway. Thus, overexpression of IFS2 can increase isoflavone mass fraction.

## CONCLUSIONS

In this study, the Bragg genotype was found to have the highest isoflavone content among the 17 cultivars. The genistein/daidzein mass ratio in these cultivars showed a significantly negative correlation with off-flavour-determining parameters such as carbonyl and TBA values. Due to their antioxidant effect, this inverse relationship suggests the role of isoflavones in eliminating off-flavour. Thus, a cultivar with a higher isoflavone content could provide better protection against oxidative damage. Higher IFS2 gene expression corresponds to higher isoflavone content in soybean seeds than IFS1. Cloning and *in silico* analysis of IFS1 and IFS2 showed more interactions of IFS2 with other proteins in the isoflavone biosynthesis pathway, which is supported by earlier studies in the same direction. Further investigation of the interactions and CHR, IFS isoforms, and other pathway proteins can provide new insights for the development of innovative strategies for metabolic engineering. The development of soy plants overexpressing IFS can minimise genotypic differences, allowing us to compare the variations under the conditions of high and low isoflavone contents with the same genotypic background concerning the formation of off-flavour. This can help to conclusively determine the role of isoflavones in off-flavour scavenging.

## Appendix

**Table S1:** Primer sequences used for quantitative-real time polymerase chain reaction (PCR) expression analysis and the cDNA cloning of isoflavone synthase IFS1 and IFS2 genes

**Gene**	**Primer**		**Sequence**
**IFS1-qRT**	F		AGAATTCCGTCCCGAGAGGTT
	R		TGCCATTCCTGAAGTAGCCAA
**IFS2-qRT**	F		AATGTGCCCTGGAGTCAATCTG
	R		GGCGTCACCACCCTTCAATAT
**Actin 2/7-qRT**	F		ACTTGCCCATCAGGAAGCTC
	R		TGTTCACCACCTCTGCCAAG
**IFS1**	F		CACCATGTTGCTGGAACTTGCACTTGGT
	R		TTAAGAAAGGAGTTTAGATGCAACGCC
**IFS2**	F		CACCATGTTGCTTGAACTTGCACTTGGT
	R		TTAAGAAAGGAGTTTAGATGCAACGCC

## References

[1] Qin P Wang T Luo Y. A review on plant-based proteins from soybean: Health benefits and soy product development. J Agric Food Res. 2022;7:100265. 10.1016/j.jafr.2021.100265

[2] Hughes GJ Ryan DJ Mukherjea R Schasteen CS. Protein digestibility-corrected amino acid scores (PDCAAS) for soy protein isolates and concentrate: Criteria for evaluation. J Agric Food Chem. 2011;59(23):12707–12. 10.1021/jf203220v 22017752

[3] Sasi M Kumar S Hasan M Arpitha SR Garcia-Gutierrez E Kumari S Current trends in the development of soy-based foods containing probiotics and paving the path for soy-synbiotics. Crit Rev Food Sci Nutr. 2023;63(29):9995–10013. 10.1080/10408398.2022.2078272 35611888

[4] Song JH Shin G Kim HJ Lee SB Moon JY Jeong JC Mutation of *GmIPK1* gene using CRISPR/Cas9 reduced phytic acid content in soybean seeds. Int J Mol Sci. 2022;23(18):10583. 10.3390/ijms231810583 PMC9504718 36142495

[5] DeMers LC Raboy V Li S Saghai Maroof MA. Network inference of transcriptional regulation in germinating low phytic acid soybean seeds. Front Plant Sci. 2021;12:708286. 10.3389/fpls.2021.708286 34531883 PMC8438133

[6] Nedele AK Gross S Rigling M Zhang Y. Reduction of green off-flavor compounds: Comparison of key odorants during fermentation of soy drink with *Lycoperdon pyriforme*. Food Chem. 2021;334:127591. 10.1016/j.foodchem.2020.127591 32721838

[7] Kumar V Rani A Rawal R. First Indian soybean variety free from off-flavour generating lipoxygenase-2 gene identified for release for commercial cultivation. Natl Acad Sci Lett. 2021;44(6):477–80. 10.1007/s40009-021-01046-x

[8] Tsen SY Tan XY Tan YM Yan BY Loke WM. Relative inhibitions of 5-lipoxygenase and myeloperoxidase and free-radical scavenging activities of daidzein, dihydrodaidzein, and equol. J Med Food. 2016;19(6):543–8. 10.1089/jmf.2015.3557 27027338

[9] Dahuja A Madaan TR. Off-flavour development in soybeans: Comparative role of some antioxidants and related enzymes. J Sci Food Agric. 2004;84(6):547–50. 10.1002/jsfa.1667

[10] Abotaleb M Samuel SM Varghese E Varghese S Kubatka P Liskova A Flavonoids in cancer and apoptosis. Cancers (Basel). 2018;11(1):28. 10.3390/cancers11010028 30597838 PMC6357032

[11] Křížová L Dadáková K Kašparovská J Kašparovský T. Isoflavones. Molecules. 2019;24(6):1076. 10.3390/molecules24061076 30893792 PMC6470817

[12] Yamagata K. Soy isoflavones inhibit endothelial cell dysfunction and prevent cardiovascular disease. J Cardiovasc Pharmacol. 2019;74(3):201–9. 10.1097/FJC.0000000000000708 31356541

[13] Sohn SI Pandian S Oh YJ Kang HJ Cho WS Cho YS. Metabolic engineering of isoflavones: An updated overview. Front Plant Sci. 2021;12:670103. 10.3389/fpls.2021.670103 34163508 PMC8216759

[14] Jung W Yu O Lau SM O’Keefe DP Odell J Fader G Identification and expression of isoflavone synthase, the key enzyme for biosynthesis of isoflavones in legumes. Nat Biotechnol. 2000;18(2):208–12. 10.1038/72671 10657130

[15] Gutierrez-Gonzalez JJ Guttikonda SK Tran LSP Aldrich DL Zhong R Yu O Differential expression of isoflavone biosynthetic genes in soybean during water deficits. Plant Cell Physiol. 2010;51(6):936–48. 10.1093/pcp/pcq065 20430761

[16] Mahesha HG Singh SA Rao AGA. Inhibition of lipoxygenase by soy isoflavones: Evidence of isoflavones as redox inhibitors. Arch Biochem Biophys. 2007;461(2):176–85. 10.1016/j.abb.2007.02.013 17391639

[17] Tewari K Kumari S Vinutha T Singh B Dahuja A. Gamma irradiation induces reduction in the off-flavour generation in soybean through enhancement of its antioxidant potential. J Radioanal Nucl Chem. 2014;303(3):2041–51. 10.1007/s10967-014-3803-9

[18] Kumar V Rani A Dixit AK Pratap D Bhatnagar D. A comparative assessment of total phenolic content, ferric reducing-anti-oxidative power, free radical-scavenging activity, vitamin C and isoflavones content in soybean with varying seed coat colour. Food Res Int. 2010;43(1):323–8. 10.1016/j.foodres.2009.10.019

[19] Ohkawa H Ohishi N Yagi K. Assay for lipid peroxides in animal tissues by thiobarbituric acid reaction. Anal Biochem. 1979;95(2):351–8. 10.1016/0003-2697(79)90738-3 36810

[20] Henick AS Benca MF Mitchell JH. Estimating carbonyl compounds in rancid fats and foods. J Am Oil Chem Soc. 1954;31(3):88–91. 10.1007/BF02612488

[21] Chomczynski P Sacchi N. Single-step method of RNA isolation by acid guanidinium thiocyanate-phenol-chloroform extraction. Anal Biochem. 1987;162(1):156–9. 10.1016/0003-2697(87)90021-2 2440339

[22] Ye J Coulouris G Zaretskaya I Cutcutache I Rozen S Madden TL. Primer-BLAST: A tool to design target-specific primers for polymerase chain reaction. BMC Bioinformatics. 2012;13:134. 10.1186/1471-2105-13-134 22708584 PMC3412702

[23] Livak KJ Schmittgen TD. Analysis of relative gene expression data using real-time quantitative PCR and the 2^−ΔΔCT^ method. Methods. 2001;25(4):402–8. 10.1006/meth.2001.1262 11846609

[24] Altschul SF Madden TL Schäffer AA Zhang J Zhang Z Miller W Gapped BLAST and PSI-BLAST: A new generation of protein database search programs. Nucleic Acids Res. 1997;25(17):3389–402. 10.1093/nar/25.17.3389 9254694 PMC146917

[25] Baek M DiMaio F Anishchenko I Dauparas J Ovchinnikov S Lee GR Accurate prediction of protein structures and interactions using a three-track neural network. Science. 2021;373(6557):871–6. 10.1126/science.abj8754 34282049 PMC7612213

[26] Ramachandran GN Ramakrishnan C Sasisekharan V. Stereochemistry of polypeptide chain configurations. J Mol Biol. 1963;7(1):95–9. 10.1016/S0022-2836(63)80023-6 13990617

[27] Szklarczyk D Franceschini A Wyder S Forslund K Heller D Huerta-Cepas J STRING v10: Protein-protein interaction networks, integrated over the tree of life. Nucleic Acids Res. 2015;43 D1:D447–52. 10.1093/nar/gku1003 25352553 PMC4383874

[28] Azam M Zhang S Abdelghany AM Shaibu AS Feng Y Li Y Seed isoflavone profiling of 1168 soybean accessions from major growing ecoregions in China. Food Res Int. 2020;130:108957. 10.1016/j.foodres.2019.108957 32156396

[29] Tepavčević V Cvejić J Poša M Bjelica A Miladinović J Rizou M Classification and discrimination of soybean (*Glycine max* (L.) Merr.) genotypes based on their isoflavone content. J Food Compos Anal. 2021;95:103670. 10.1016/j.jfca.2020.103670

[30] de Queirós LD de Ávila ARA Botaro AV Chirotto DBL Macedo JA Macedo GA. Combined isoflavones biotransformation increases the bioactive and antioxidant capacity of soymilk. Appl Microbiol Biotechnol. 2020;104(23):10019–31. 10.1007/s00253-020-10986-1 33136177

[31] Ganhão R Estévez M Morcuende D. Suitability of the TBA method for assessing lipid oxidation in a meat system with added phenolic-rich materials. Food Chem. 2011;126(2):772–8. 10.1016/j.foodchem.2010.11.064

[32] Ruiz-Larrea MB Mohan AR Paganga G Miller NJ Bolwell GP Rice-Evans CA. Antioxidant activity of phytoestrogenic isoflavones. Free Radic Res. 1997;26(1):63–70. 10.3109/10715769709097785 9018473

[33] Vicaş SI Chedea VS Socaciu C. Inhibitory effects of isoflavones on soybean lipoxygenase-1 activity. J Food Biochem. 2011;35(2):613–27. 10.1111/j.1745-4514.2010.00405.x

[34] Liu S Grierson D Xi W. Biosynthesis, distribution, nutritional and organoleptic properties of bitter compounds in fruit and vegetables. Crit Rev Food Sci Nutr. 2022;62:1–20. 10.1080/10408398.2022.2119930 36099178

[35] Roland WSU Vincken JP Gouka RJ van Buren L Gruppen H Smit G. Soy isoflavones and other isoflavonoids activate the human bitter taste receptors hTAS2R14 and hTAS2R39. J Agric Food Chem. 2011;59(21):11764–71. 10.1021/jf202816u 21942422

[36] Drewnowski A Gomez-Carneros C. Bitter taste, phytonutrients, and the consumer: A review. Am J Clin Nutr. 2000;72(6):1424–35. 10.1093/ajcn/72.6.1424 11101467

[37] Dhaubhadel S McGarvey BD Williams R Gijzen M. Isoflavonoid biosynthesis and accumulation in developing soybean seeds. Plant Mol Biol. 2003;53(6):733–43. 10.1023/B:PLAN.0000023666.30358.ae 15082922

[38] Dastmalchi M Bernards MA Dhaubhadel S. Twin anchors of the soybean isoflavonoid metabolon: Evidence for tethering of the complex to the endoplasmic reticulum by IFS and C4H. Plant J. 2016;85(6):689–706. 10.1111/tpj.13137 26856401

[39] Dinkins RD Hancock J Coe BL May JB Goodman JP Bass WT Isoflavone levels, nodulation and gene expression profiles of a CRISPR/Cas9 deletion mutant in the isoflavone synthase gene of red clover. Plant Cell Rep. 2021;40(3):517–28. 10.1007/s00299-020-02647-4 33389047

